# Pulsed electromagnetic fields reduce acute inflammation in the injured rat‐tail intervertebral disc

**DOI:** 10.1002/jsp2.1069

**Published:** 2019-12-02

**Authors:** Andrew K. Chan, Xinyan Tang, Nikhil V. Mummaneni, Dezba Coughlin, Ellen Liebenberg, Annie Ouyang, Stefan Dudli, Michael Lauricella, Nianli Zhang, Erik I. Waldorff, James T. Ryaby, Jeffrey C. Lotz

**Affiliations:** ^1^ Department of Neurological Surgery University of California San Francisco San Francisco California; ^2^ Department of Orthopaedic Surgery University of California San Francisco California; ^3^ Orthofix Inc. Lewisville Texas

**Keywords:** degeneration model, inflammation, needle stab, pulsed electromagnetic fields, rat‐tail disc

## Abstract

Pro‐inflammatory cytokines are recognized contributors to intervertebral disc (IVD) degeneration and discogenic pain. We have recently reported the anti‐inflammatory effect of pulsed electromagnetic fields (PEMF) on IVD cells in vitro. Whether these potentially therapeutic effects are sufficiently potent to influence disc health in vivo has not been demonstrated. We report here the effect of PEMF on acute inflammation arising from a rat‐tail IVD injury model. Disc degeneration was induced by percutaneously stabbing the Co6‐7, Co7‐8, and Co8‐9 levels using a 20‐gauge needle. Seventy‐two (72) rats were divided into three groups: sham control, needle stab, needle stab+PEMF. Treated rats were exposed to PEMF immediately following surgery and for either 4 or 7 days (4 hr/d). Stab and PEMF effects were evaluated by measuring inflammatory cytokine gene expression (RT‐PCR) and protein levels (ELISA assay), anabolic and catabolic gene expression (RT‐PCR), and histologic changes. We observed in untreated animals that at day 7 after injury, inflammatory cytokines (interleukin [IL]‐6, tumor necrosis factor α, and IL‐1β) were significantly increased at both gene and protein levels (*P* < .05). Similarly, catabolic factors (MMP [metalloproteinases]‐2, MMP‐13 and the transcriptional factor NF‐kβ gene expression) were significantly increased (*P* < .05). At day 7, PEMF treatment significantly inhibited inflammatory cytokine gene and protein expression induced by needle stab injury (*P* < .05). At day 4, PEMF downregulated FGF‐1 and upregulated MMP‐2 compared to the stab‐only group. These data demonstrate that previously reported anti‐inflammatory effects of PEMF on disc cells carry over to the in vivo situation, suggesting potential therapeutic benefits. Though we observed an inhibitory effect of PEMF on acute inflammatory cytokine expression, a consistent effect was not observed for acute changes in disc histology and anabolic and catabolic factor expression. Therefore, these findings should be further investigated in studies of longer duration following needle‐stab injury.

## Graphical abstract

Intervertebral Disc Degeneration Model: Rat‐Tail Needle Stab. Pulsed electromagnetic fieldtreatment was associated with a decrease in inflammatory cytokine gene expression in the rat disc 7 days following injury

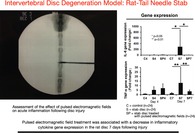

## 1 INTRODUCTION

Back pain‐related disabilities are commonly attributed to intervertebral disc (IVD) degeneration.^1^ There is increasing evidence that symptomatic disc degeneration associates with elevated levels of pro‐inflammatory cytokines, such as those from the interleukin family (IL‐1, IL‐2, IL‐6, IL‐8, and IL‐17), interferon gamma (IFN‐ɤ), and tumor necrosis factor (TNF‐α).^2^, ^3^, ^4^ These cytokines contribute to a proinflammatory feedback loop that includes immune cell activation and infiltration,^5^, ^6^ catabolic degenerative processes,^7^, ^8^ interference with tissue regeneration,^3^, ^9^, ^10^, ^11^, ^12^ and ultimately lead to changes in disc structure,^13^ discogenic back pain, and disc herniation.^14^, ^15^, ^16^ Non‐invasive treatments that target these cytokines have been pursued as nonsurgical options to relieve degeneration‐associated symptoms.^17^, ^18^, ^19^

Pulsed electromagnetic fields (PEMF) are a noninvasive, biophysical stimulus that has been used for improving the success rate of spine fusion, as well as treating pseudarthrosis and osteoporosis.^20^, ^21^ PEMF were first introduced as an alternative^22^ to surgically implanted electrodes in bone^23^ for the treatment of fracture nonunions.^24^ The clinical device employs a time‐varying electromagnetic field that is delivered via an inductive antenna toward a target tissue^25^ with differential therapeutic effects depending on the applied waveform characteristics (eg, amplitude, duration, and frequency).^26^, ^27^, ^28^, ^29^ The FDA‐approved waveforms for clinical use are designed to penetrate thorough human tissue in order to deliver noninvasive therapy. The specific mechanisms underlying the effect of PEMF on cellular and biophysical properties remains an area of ongoing research. Described mechanisms of effect include increased calcium ion signaling,^30^, ^31^ induction of the gaseous signaling molecule nitric oxide,^29^, ^31^, ^32^, ^33^ increase in the expression of heat shock proteins,^34^, ^35^ and increased expression of cell membrane adenosine receptors.^36^ The breadth of proposed mechanisms underscores the likely multiple biological cascades that PEMF exerts therapeutic effects.

Recent studies demonstrate that PEMF can promote cartilage and bone metabolism by stimulation of cell proliferation/differentiation and matrix synthesis.^37^, ^38^, ^39^ Moreover, PEMF may protect cartilage and surrounding tissues from a detrimental and catabolic environment associated with osteoarthritis^40^, ^41^ by inhibiting pro‐inflammatory cytokine release from human fibroblasts and chondrocytes.^42^, ^43^ Accordingly, PEMF stimulation has been employed to treat pain, inflammation, and dysfunction associated with rheumatoid arthritis (RA) and osteoarthritis (OA).^44^

PEMF may also have a potential to treat low back pain secondary to IVD degeneration. Our recent study demonstrates that PEMF treatment leads to a reduction in the expression of genes associated with IVD degeneration in human IVD cells *in vitro*.^45^ However, the application to IVD degeneration has yet to be evaluated in vivo. To test whether in vitro responses carry over to the in vivo situation, we utilized the IVD needle puncture model, which has been shown to trigger disc degeneration in multiple animal models, and results in an alteration of disc morphology, unique biochemical profiles, and an acute inflammatory cascade characteristic of human degenerative discs.^46^, ^47^, ^48^ The rat‐tail needle puncture is a common model owing to a repeatable, well‐characterized post‐injury response.^49^, ^50^, ^51^, ^52^, ^53^ Consequently, the objective of the present study was to conduct the first in vivo trial of PEMF as a therapy for disc injury‐associated inflammation. The results will help to clarify PEMF disease modifying activities mediated by differential inflammatory responses, and thus indicate whether PEMF holds the potential to improve back pain in patients with IVD degeneration.

## 2 METHODS

### 2.1 Ethics statement

All experimental procedures were approved by institutional animal care and use committee at the University of California San Francisco (UCSF) (protocol number: AN108985‐01F).

### 2.2 Animals

Seventy‐two, 3‐ to 4‐month‐old female Sprague‐Dawley rats were used in this study (weight: 265‐310 g, from Lab Animal Resource Center, UCSF). Water and food were provided without restrictions. Temperatures were maintained at 24 **°**C. Light schedules included 12 hours daylight starting at 7:00 am and 12 hours darkness start at 7:00 pm.

### 2.3 Surgical protocol

Rats were anesthetized using 1.5% isoflurane (IsoFlo, ISoflurane, USP, Abbott Laboratories, North Chicago, Illinois) in a chamber and monitored by an assistant during surgery, then immediately thereafter, received an intraperitoneal injection of analgesic buprenorphine (0.01 mg/kg, SQ). Proper depth of anesthesia was assessed by monitoring of hind paw pinch and respiration rate. A constant body temperature of 37 **°**C was maintained with a heating pad.

**Figure 1:**
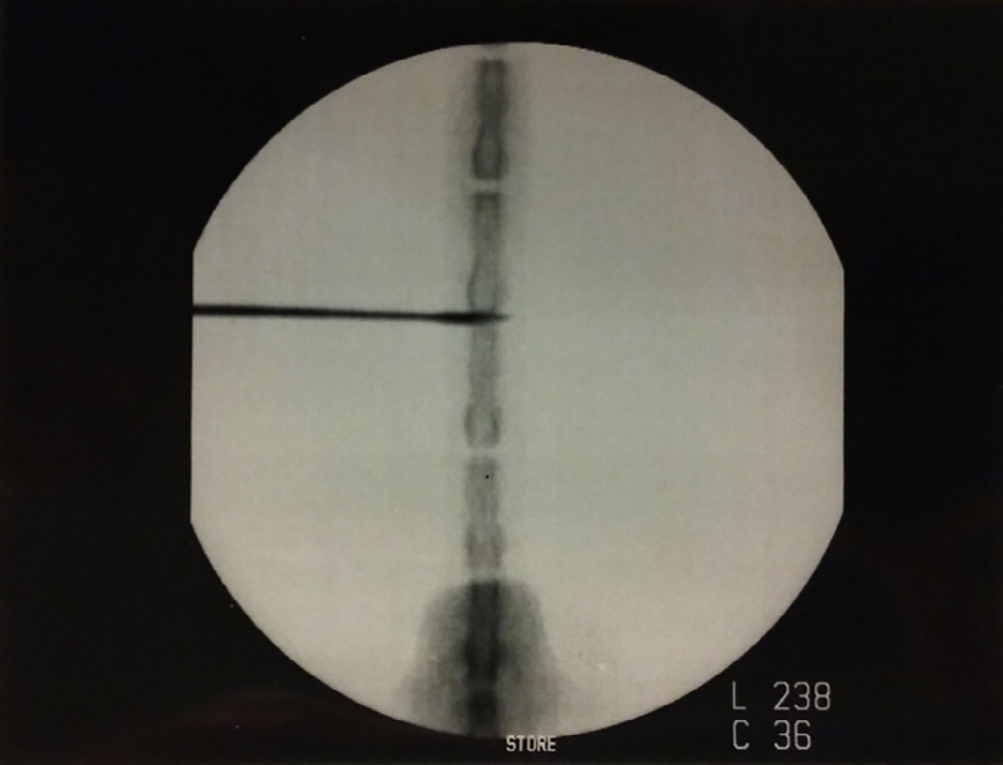
Rat caudal disc were stabbed by a 20‐gauge needle under the guidance of C‐arm

The rats were randomly divided into three groups: sham control, C, (n = 24), needle‐stab injury group, S, (n = 24), and needle stab injury plus PEMF treatment group, SP, (n = 24). All surgical procedures were performed under aseptic conditions. After tail skin Betadine (Betadine, surgical scrub, Povidone‐iodine, 7.5%, Purdue Products, L.P., Stamford, Connecticut) and alcohol prep, a 20‐gauge needle (Becton Dickinson, New Jersey) was stabbed into the rat tail disc at Co6‐Co7, Co7‐Co8, and Co8‐Co9 levels under a C‐arm for fluoroscopic navigating (OEC Medical Systems Inc., Germany) for the needle stab injury group (n = 24) and the needle stab injury plus PEMF group (n = 24) (Figure 1). The needle puncture technique was in accordance to previous studies.^49^, ^50^, ^51^, ^52^, ^53^, ^54^, ^55^, ^56^ Specifically, the needle was inserted and passed through the nucleus pulposus up to the deep contralateral annulus fibrosus. Then, the needle was rotated 180^°^ before removal. After surgery, Betadine gauze was used to clean the stab sites. After surgery, the rats were allowed to recover on a heating pad in a recovery chamber before returning to their cages. The experimental tail discs were labeled with a permanent skin marker. For the sham control group (n = 24), rats were placed under anesthesia but no needle stab injury was performed.

### 2.4 PEMF treatment

**Figure 2:**
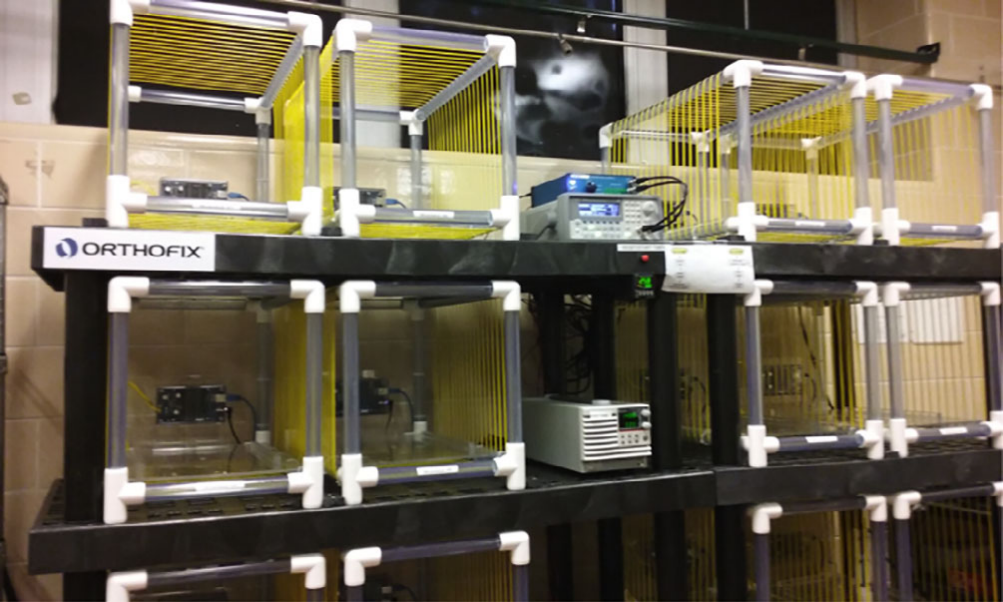
Dial‐A‐Stim IV in vivo Rat System

For the rats assigned to the PEMF treatment group, PEMF was applied via a custom PEMF rat cage (Dial‐A‐Stim IV in vivo Rat System), which includes an integrated electrical coil powered by a direct current power source. It delivers a PEMF waveform similar to the clinically‐approved Physio‐Stim PEMF device (Orthofix Inc., Lewisville, Texas), which consists of a square wave with a 25% duty cycle and 3.846 kHz frequency. A dB/dt sensor was used to verify the magnetic field in each cage prior to each PEMF treatment (Figure 2). Napa Nectar water gel packs (Systems Engineering, NAPA, California) were provided as a nonmagnetic water bottle substitute. The rats in the PEMF treatment group commenced PEMF treatment (4 hr/d) immediately following surgery until sacrifice.

### 2.5 Specimen harvest

The rats were euthanized by carbon dioxide asphyxiation overdose followed by bilateral thoracotomy. The Co6/Co7 disc—including endplate and bone—were cut out and fixed in 10% neutral buffered formalin (BDH) for histology staining; the Co7/Co8 disc was collected and snap frozen in liquid nitrogen immediately and then stored at −80 °C until RNA extraction; the Co8/Co9 disc was harvested and stored at −80 °C for protein assay. Harvesting was conducted on day 4 (n = 36, 12 from each treatment condition) and day 7 (n = 36, 12 from each treatment condition) postoperatively.

### 2.6 Real time RT‐PCR

Disc tissue samples—including both NP and AF tissue—were homogenized in Trizol reagent (Life Technology, California) with a Tissue Raptor (Qiagen), and followed by standard procedure for RNA isolation and cDNA synthesis. Real time PCR was performed with an iCycler iQ system (BioRad, Hercules, California). β‐actin was selected as the internal reference. Twelve bio‐replicates with three technical replicates per group for PCR reaction were utilized. Inflammatory cytokine genes, interleukin 6 (IL‐6), interleukin 1β (IL‐β), tumor necrosis factor α (TNF‐α), and anabolic genes fibroblast growth factor (FGF‐1), hyaluronan and proteoglycan binding link protein (HAPLN‐1), and catabolic genes for matrix metalloproteinases (MMP2, MMP13) and NF‐kβ were examined.

### 2.7 Enzyme‐linked immunosorbent assay (ELISA assay)

The tissue samples (including both NP and AF tissue) were homogenized and extracted with TRETissue protein extraction reagent (Thermo Scientific), and total protein was quantified with Pierce BCA protein assay (Thermo Scientific). Samples were harvested from a single cut whole rat‐tail disc, which was placed in its entirety in a 1.5‐mL Eppendorf tube. The tissue was stored in a −80 °C freezer until homogenization and analysis. The volume to weight ratio of extraction buffer to disc tissue was 1 g of tissue to 20 mL of extraction reagent. The tissue was treated in buffer for 6 minutes at 4 °C with a bead shaking instrument. The tissue was completely dissolved after centrifuge, with little insoluble matrix as is expected. IL‐6, IL‐1β, and TNF‐α protein levels were detected with Aushon Rat multiplex cytokine ELISA assay (Aushon Biosystems, Inc. Massachusetts). Twelve bio‐replicates with two technical replicates for each group were utilized.

## 3 HISTOLOGY

After fixation in 10% neutral buffered formalin and decalcification in IED (Biocare), disc tissues were embedded in paraffin (Fisher) and cut into 7‐μm‐thick sagittal sections through the disc—parallel to the direction of the stab—and stained with Safranin O, Fast Green and Hematoxylin. Histological images were acquired with a light microscope (Nikon Eclipse E800). The staining was graded using a standard scoring scale established previously to assess the cellular and morphologic changes in both annulus fibrosis and nucleus pulposus (total score range 6 [least disruption]−18 [most disruption] points) (Table 1).^48^, ^57^ Safranin O‐Fast Green staining was graded based on the scale described.^51^, ^58^ The six components scored included disc morphology (AF and NP), NP cellularity, matrix structure, cartilage endplate interruption, and infiltration. For each component, scales ranged from normal (Grade 1) to severe disruption (Grade 3; Table 1). These were rated by two researchers blinded to the treatment group allocations.

**Table 1:** Histologic rating scale (total: 6‐18 points)

**Annulus organization**
1. Normal
2. Disrupted pattern <30%
3. Disrupted pattern >30%
**Cartilage endplate**
1. Normal
2. Minimally interrupted
3. Moderate/severe interruption
**Cellularity of nucleus**
1. Normal: notochordal cells distributed throughout matrix
2. Moderate decrease in cellularity
3. Severe decrease in cellularity
**Matrix structure in nucleus**
1. Cells distributed individually in lacey matrix or small clumps of notochordal cells in amorphous matrix
2. Large clumps of cells in amorphous matrix
3. Amorphous matrix with few or no clumps of cells
**Size and shape of nucleus**
1. Normal: large oval
2. Nucleus reduced in size <50%
3. Nucleus reduced>50%
**Cellular infiltrate** a
1. Absent
2. Some
3. Extensive

a Refers to infiltration of immune cells during the inflammatory reaction to disc injury.

## 4 STATISTICAL ANALYSIS

All descriptive statistics are reported as mean values ± S.D. Statistical differences were tested using a one‐way analysis of variance (anova), followed by Tukey HSD for comparing multiple groups. All analyses were performed using StatView 5.0 (SAS institute, Inc. Cary, North Carolina). *P* values less than .05 were considered to be statistically significant.

## 5 RESULTS

The surgical procedure was well tolerated and no intraoperative complications were encountered.

## 6 PEMF EFFECTS ON IVD GENE EXPRESSION AND PROTEIN LEVELS

### 6.1 Inflammatory mediators

#### 6.1.1 IL‐6 gene and protein expression

**Figure 3:**
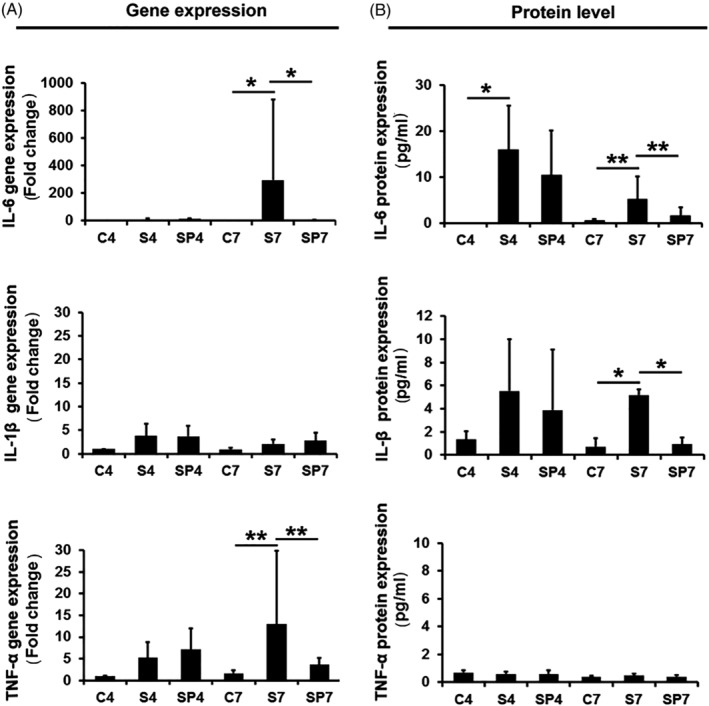
PEMF treatment effects on inflammatory cytokine gene expression and protein expression. A, Gene expression. B, Protein level. C, sham control (n = 24); S, needle stab (n = 24), SP, needle stab+PEMF (n = 24). Numbers indicate day 4 and day 7. Error bars represent one SD. * represents *P* < .05, ** represents *P* < .01

IL‐6 expression was very low in the sham control group at both the gene and protein level. However, IL‐6 expression increased dramatically in the stab‐injury group at the gene level on day 7 (293.3‐fold change [Il‐6 treatment/control]) and protein level on both day 4 (15.9 ± 9.6 pg/mL vs 0 pg/mL) and day 7 (5.2 ± 4.9 pg/mL vs 0.5 ± 0.3 pg/mL) (*P* < .05; Figure 3A,B). There was no significant difference for IL‐6 gene expression on day 4.

PEMF treatment significantly reduced IL‐6 gene expression (from 293.3‐fold change to 2.93‐fold change [IL‐6 treatment/control]; *P* < .05) and IL‐6 protein expression (from 5.2 ± 4.9 to 1.6 ± 1.9 pg/mL; *P* < .01) at day 7 (Figure 3A,B). However, at day 4, PEMF treatment did not exert statistically significant inhibitory effects on IL‐6 gene or protein expression (*P* > .05).

#### 6.1.2 IL‐1β gene and protein expression

Similar to IL‐6, IL‐1β was expressed at a very low level in the sham control group for both gene and protein expression on both day 4 and 7. In contrast, to IL‐6, needle stab triggered only a slight elevation of IL‐1β gene expression on day 4 and day 7 (3.7‐ and 2.1‐fold increase, respectively; *P* > .05). However, needle stab dramatically increased IL‐1β protein levels at day 7 (from 0.7 ± 0.7 to 5.2 ± 0.5 pg/mL; *P* < .05), which was significantly reduced by PEMF treatment (from 5.2 ± 0.5 to 0.9 ± 0.6 pg/mL; *P* < .05) (Figure 3A,B). While similar trends were observed on day 4, the differences did not reach statistical significance.

#### 6.1.3 TNF‐α gene and protein expression

TNF‐α was expressed at only a low level in the sham control group, with needle stab significantly increasing gene expression at day 7 (13.0‐fold change TNF‐α stab/control expression; *P* < .01). This was significantly reduced by PEMF treatment (from 13.0‐fold change to 3.6‐fold change TNF‐α stab/control expression; *P* < .01) (Figure 3A). There were no significant differences in gene expression at day 4 between sham control, needle stab, and PEMF treatment groups. Additionally, as distinct from IL‐1β, there were no measurable differences for TNF‐α protein level expression between the sham control, stab, and PEMF treatment groups at both days 4 and 7.

### 6.2 Anabolic and catabolic gene expression

#### 6.2.1 FGF‐1 gene expression

**Figure 4:**
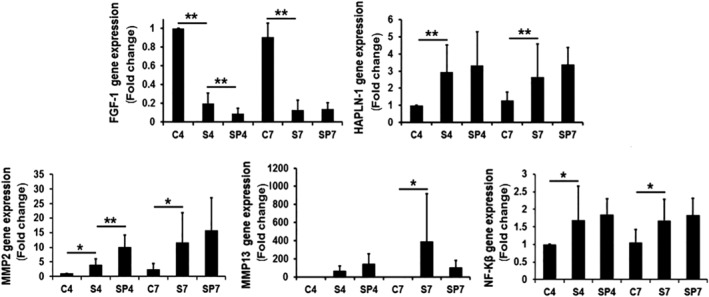
PEMF treatment effects on anabolic and catabolic gene expression. Error bars represent one SD. * *P* < .05, ** represents *P* < .01. C, sham control (n = 24); S, needle stab (n = 24); SP, needle stab+PEMF (n = 24). Numbers indicate day 4 and day 7

FGF‐1 expression was significantly decreased in stab‐injury group compared with sham control group at both days 4 and day 7 (0.2‐fold change and 0.1‐fold change FGF‐1 stab/control, respectively; *P* < .01) (Figure 4). PEMF treatment suppressed FGF‐1 even more at day 4 compared with stab‐injury group (from 0.2‐fold change to 0.1‐fold change; *P* < .01), while no significant PEMF effect on FGF‐1 expression was observed at day 7.

#### 6.2.2 HAPLN‐1 gene expression

HAPLN‐1 expression was highly increased in the stab‐injury group at day 4 (2.9‐fold change) and day 7 (2.7‐fold change) compared to sham control (*P* < .01), and tended to further increase when treated with PEMF (Figure 4), though this result did not reach statistical significance at day 4 and 7.

#### 6.2.3 MMP2, MMP13, NF‐Kβ gene expression

In the stab injury group, MMP2 expression was significantly increased at both day 4 and day 7 (4.0‐fold change and 11.7‐fold change, respectively; *P* < .05; Figure 4) compared to the sham control. PEMF treatment further increased MMP2 expression compared with the stab‐injury group at day 4 (from 4.0 to 10.1‐fold change; *P* < .01). A similar increase was observed on day 7 though the result did not reach statistical significance (*P* > .05).

MMP13 expression was induced by needle stab at day 7 (390.9‐fold change; *P* < .05). PEMF treatment tended to decrease MMP13 expression compared to the stab‐injury group at day 7, though this result did not reach significance. There was no significant difference for MMP13 gene expression on day 4 between sham control, stab injury, or PEMF groups.

NF‐Kβ expression was significantly increased by needle stab at day 4 (1.7‐fold change; *P* < .05) and day 7 (1.7‐fold change; *P* < .05) compared to the sham control. Though PEMF treatment induced more NF‐Kβ expression compared to the needle‐stab only group, these results did not reach statistical significance at day 4 and 7 (*P* > .05; Figure 4).

## 7 HISTOLOGIC EVALUATIONS

**Figure 5:**
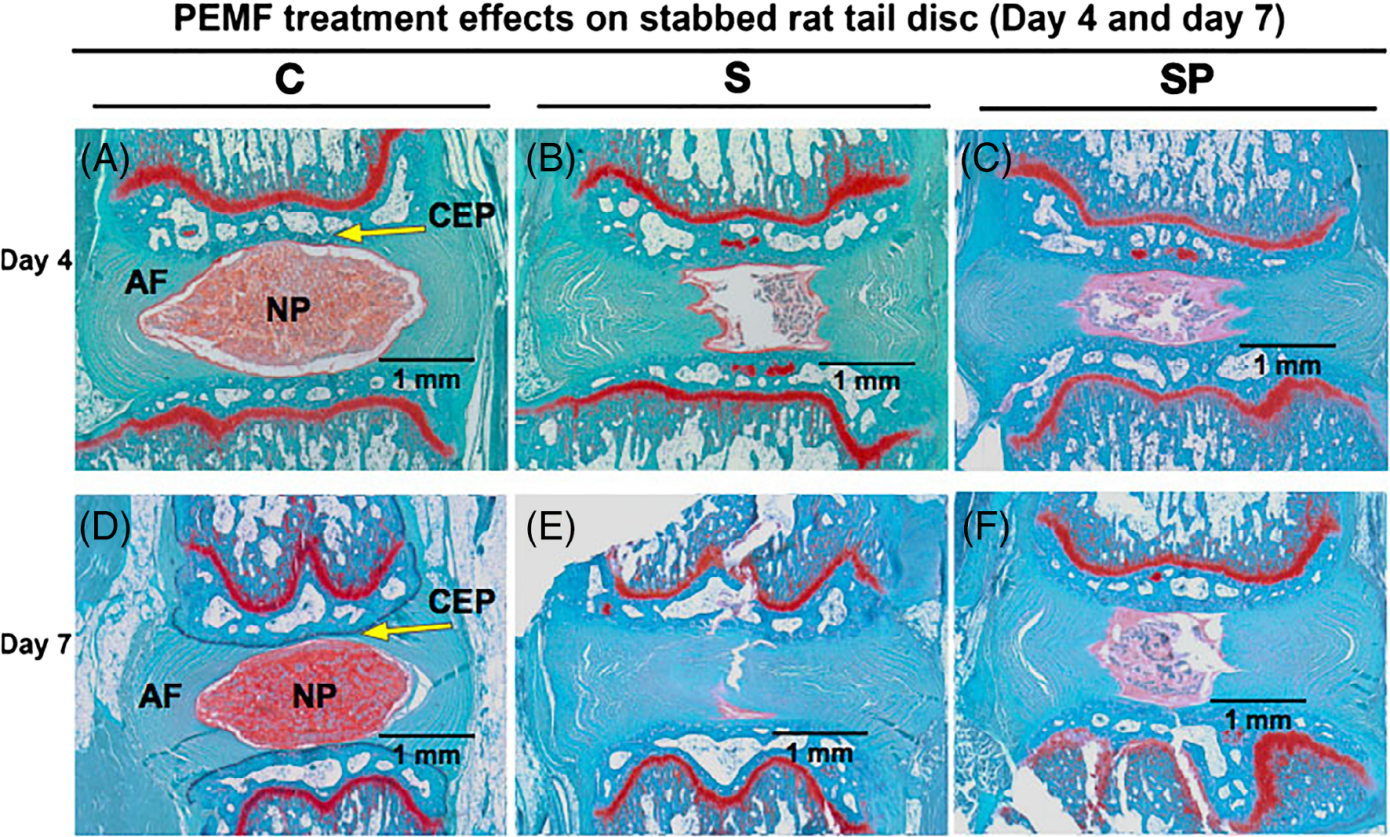
Representative histologic appearance of rat caudal disc at day 4 and 7 after needle stab and PEMF treatment. Safranin O staining showing matrix production and nucleus pulposus structure; counterstained with Fast Green. A, Day 4 sham control (C). B, Day 4 needle stab (S). C, Day 4 needle stab+PEMF (SP). D, Day 7 sham control (C). E, Day 7 needle stab (S). F, Day 7 needle stab +PEMF (SP). AF, annulus fibrosus; NP, nucleus pulposus; CEP, cartilage endplate

**Figure 6:**
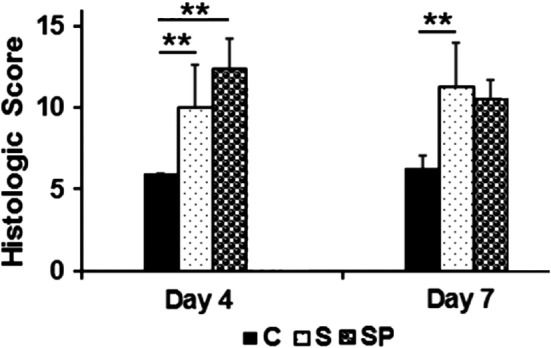
Histologic score of disc in sham control group, needle stab group and needle stab+ PEMF group. Histologic grading is based on the six categories in Table 1, and classified into three grades: normal (grade 1), moderate degeneration (grade 2), and severe degeneration (grade 3). Overall grades for a normal disc and the most severely degenerated disc would be assigned grades of 6 and 18, respectively. **P < .01. C, control (n = 24); S, stab (n = 24); SP, stab + PEMF (n = 24)

The discs were categorized as normal (Grade 1), moderately degenerated (Grade 2) and severely degenerated (Grade 3) using a common grading scheme (Table 1). Average overall scores and subcategory scores for each cohort are provided in Table 2. The control group showed normal morphology and structure including oval nucleus pulposus (NP), evenly distributed cells, well‐organized annulus fibrosus (AF) without inward protrusion of fiber lamellae, intact cartilage endplate, and a clear AF‐NP boundary (Figure 5A,D). In the needle‐stab group, NP revealed an irregular shape, decreased cellularity, disorganized and inward‐protruded fiber lamellae in AF, disrupted endplate (partially) and unclear AF‐NP boundary (Figure 5B,E). In the PEMF treatment group, AF lamellae appeared less disorganized than for needle‐stab group at both day 4 and day 7 (Figure 5C,F) (to demonstrate the extent of histologic variability, examples of worst, average, and best histologic specimens for the needle‐stab and PEMF treatment groups—at both day 4 and day 7—are available in Figure S1; control specimens were homogeneous histologically and were not included in this supplemental analysis). Still, these qualitative differences were not supported by the quantitative histologic rating score analysis (Figure 6). Though the histologic rating score showed significant changes between control group and needle‐stab groups at both day 4 and day 7 (*P* < .01), no significant changes were noted in PEMF treated groups compared with needle‐stab group—although there was a decreasing trend at day 7 in the PEMF group.

**Table 2:** Average Histologic Rating Scale Scores

	Day 4			Day 7		
Histologic category	C	S	SP	C	S	SP
Annulus organization	1 ± 0	2.3 ± 0.6*	2.6 ± 0.5*	1.1 ± 0.4	2.7 ± 0.4*	2.5 ± 0.4*
Cartilage endplate	1 ± 0	1.2 ± 0.5	1.3 ± 0.6	1 ± 0	1.2 ± 0.3	1 ± 0
Cellularity of nucleus	1 ± 0	1.3 ± 0.6**	2.0 ± 0.5* ^,^ **	1 ± 0	1.8 ± 0.9*	2 ± 0.4*
Matrix structure in nucleus	1 ± 0	1.3 ± 0.6**	1.8 ± 0.5* ^,^ **	1.0 ± 0.1	1.9 ± 0.8*	2.0 ± 0.5*
Size and shape of nucleus	1 ± 0	1.8 ± 0.7*	2.3 ± 0.5*	1.1 ± 0.3	2.3 ± 0.7*	2.4 ± 0.4*
Cellular infiltrate	1 ± 0	1.4 ± 0.8	1.0 ± 0.1	1 ± 0	1.5 ± 0.7*	1.1 ± 0.3
Total histological score	6 ± 0	10.0 ± 2.7*	11.1 ± 1.9*	6.3 ± 0.7	11.3 ± 2.6*	10.5 ± 1.1*

* *P* < .05 for comparison with control.

** *P* < .05 for S and SP comparison.

## 8 DISCUSSION

We have previously shown that PEMF can significantly reduce pro‐inflammatory cytokine expression in human and bovine disc cells in vitro.^45^, ^59^ Similarly, others have demonstrated that PEMF exposure exerts anti‐inflammatory activity in several additional musculoskeletal cell types, such as chondrocytes, fibroblasts, tendon cells and osteoblasts in vitro.^37^, ^40^, ^42^, ^43^

The goal of the present study was to determine if the in vitro anti‐inflammatory effects were sufficiently potent to influence the in vivo situation via an analysis of (a) inflammatory, (b) anabolic and catabolic, and (c) histologic markers in a rat model of IVD degeneration.

### 8.1 PEMF blunts acute inflammatory marker expression

We demonstrate here that disc degeneration induced by a needle stab‐injury rat model caused an acute inflammatory response, associated with increased levels of pro‐inflammatory cytokines, IL‐6, IL‐1β, and TNF‐α—particularly at day 7—and is consistent with previous investigations.^3^, ^14^, ^15^, ^16^, ^52^ Furthermore, we demonstrate that PEMF can blunt these acute inflammatory effects. Specifically at day 7, PEMF treatment significantly inhibited injury‐associated IL‐6 and TNF‐α expression at the gene level as well as IL‐6 and IL‐1β expression at the protein level. Therefore, it appears that the anti‐inflammatory effect of PEMF observed in vitro can be recapitulated in vivo.

The lack of significant differences in TNF‐α protein expression may reflect the lower magnitude of TNF‐α expression—relative to IL‐1β—in both nondegenerated and degenerated IVDs and the smaller difference in TNF‐α copy number change between degenerated and nondegenerated IVD.^60^

The effect of PEMF stimulation may not be limited to down‐regulation of pro‐inflammatory cytokines. Indeed, PEMF stimulation exerts a dual effect on both upregulation of anti‐inflammatory factors^42^, ^61^ and downregulation of pro‐inflammatory factors.^42^, ^45^ For example, PEMF treatment was both associated with: (a) a reduction in pro‐inflammatory cytokines IL‐1α, IL‐1β, and TNF‐α expression; and (b) an increase in anti‐inflammatory cytokine IL‐10 expression in human fibroblast‐like cells^42^ and a cerebral ischemia mice model.^61^ These dual effects of PEMF on inflammation enable tissues to return to homeostasis, thereby promoting tissue regeneration. Taken together, these data suggest that PEMF treatment may be a potential noninvasive therapy for inflammation‐associated disc degeneration.

### 8.2 Stab injury acutely downregulates anabolic factor expression and upregulates catabolic factor expression and PEMF facilitates these changes in the acute period

Components of extracellular matrix (ECM) such as proteoglycans, glycoproteins, and collagens, are synthesized and degraded by proteases to maintain homeostasis in the IVD.^62^, ^63^, ^64^, ^65^ In the case of imbalance, excess degradation products can trigger inflammation—a process facilitated by MMP. For instance, in an in vitro study, fragmented fibronectin alleviated MMP inhibition and promoted inflammatory cell migration.^66^ Ultimately, MMPs are thought to play an important role in disc degeneration and resorption.^67^ Specifically, proinflammatory cytokines such as IL‐1 and TNF‐α induce disc degeneration by contributing to decreased matrix production and increased production of degradation enzymes such as MMPs.^63^, ^68^, ^69^, ^70^

In the acute period following needle‐stab injury, rat IVD revealed increased gene expression of MMPs (MMP2 and MMP13) in addition to catabolic NF‐kβ—consistent with prior investigation.^45^, ^70^, ^71^ Though PEMF blunted inflammatory cytokine expression following needle‐stab injury, its action did not similarly downregulate the expression of catabolic factors MMP and NF‐kβ in the acute period. In fact, MMP2 expression was facilitated by PEMF, with significantly higher levels of expression in the needle‐stab group receiving PEMF than the needle‐stab group alone. This is in contrast to our prior in vitro investigation, in which we exposed human annulus fibrosus and nucleus pulposus cells to IL‐1α and PEMF, and observed a PEMF‐associated reduction in MMP2, MMP13, and NFkB.^45^ This suggests potential species differences, or an uncoupling of PEMF's effects on inflammatory cytokine and MMP‐associated matrix breakdown in the in vivo context. Additionally, MMP2 and MMP13 belong to different families of MMPs: gelatinases and collagenases, respectively, and have unique roles in IVD degeneration.^72^ The collagenases cleave native interstitial collagen, whereas gelatinases only digest denatured collagens and nonhelical collagen in basement membranes.^72^, ^73^, ^74^ MMP‐2 in particular participates in the secondary breakdown of collagen during remodeling^70^, ^71^, ^75^, ^76^ and is critical for local matrix turnover and annulus fibrosus cell‐mediated remodeling of collagen matrix^77^ while also being implicated in the ECM destruction associated with IVD degeneration.^78^

The function of MMP in maintaining disc health is unclear given that MMP are implicated in both catabolic and tissue remodeling roles.^79^ Though specific instances of upregulation of MMP function have been associated with disc degeneration,^80^ the specific underlying MMP‐associated mechanisms involved in IVD degeneration—and the precise coordination of the molecular events underlying degenerative disc remodeling—are not well understood.^77^ Therefore, any effect of PEMF on these multiple roles remain incompletely defined and further research is needed.

In our prior in vitro investigation of PEMF, we witnessed a slight upregulation of FGF‐1 and HAPLN‐1 in IVD exposed to proinflammatory conditions. Similarly, we did observe an increased expression of HAPLN‐1 in the PEMF treated group in vivo, though the result did not reach statistical significance. HAPLN‐1 is a protein important in the formation and structural integrity of cartilaginous matrices^81^ and its upregulation may be protective in IVD degeneration.

We did not observe a similar upregulation in FGF‐1 following PEMF treatment. FGF‐1 is a protein important for chondrocyte proliferation and proteoglycan synthesis^82^, ^83^ and its loss has been linked to IVD degeneration.^82^ On the contrary, PEMF suppressed FGF‐1 at day 4. However, at day 7, after FGF‐1 gene expression further decreased in the needle‐stab only group, there was no significant difference between needle‐stab and PEMF groups. PEMF may serve to accelerate the regenerative process—as evidenced by the facilitation of FGF‐1 and MMP2 in the short term—and further investigation should investigate its impact on matrix formation and cell growth in the longer term.

### 8.3 Stab injury reveals significant acute histological changes in the IVD

Needle stab‐induced disc degeneration was also confirmed in the acute period by histological changes in disc tissue, which included altered morphology, decreased cellularity, disorganized and inward‐bulging lamellae in AF, and slightly disrupted cartilage endplate and changed proteoglycan staining. These alterations of morphology, structure, and biochemical feature recapitulate changes in degenerative discs as reported in prior investigations.^47^, ^48^, ^49^, ^50^, ^53^, ^54^, ^77^ In the acute period, PEMF treatment was not associated with significantly different overall histologic scoring changes.

Though the present investigation and others^54^ demonstrate significant histologic changes in the acute period (eg, <7 days) following needle puncture, some suggest that a longer time point (ie, at least 2‐4 weeks) may be required to demonstrate significant structural changes (eg, total collagen, glycosaminoglycan content, decreased disc height, increased catabolic factor expression, and changes in histologic and mechanical properties).^49^, ^53^ As such, the acute, 7‐day period of the present study may be insufficient to fully appreciate histopathological changes following disc injury—much less changes that may be affected by PEMF.

Though there was no significant difference in overall histological score, we did detect subtle differences in the group treated with PEMF. PEMF treatment was associated with less AF lamellae disorganization as compared to the stab‐only cohort at day 7, though this result did not reach statistical significance. This is consistent with the previously discussed changes in cell activity, and suggests a potential for PEMF to exert a beneficial effect even in the early, post‐injury period. Future, longer‐term investigation will be required to assess the effect of chronic PEMF exposure on IVD structure via an analysis of both histo‐morphological and radiographic parameters (eg, disc height).

### 8.4 Limitations

This study is not without limitations. First, the time course of the study—the acute period following disc injury—may not fully model the chronic clinical condition of disc degeneration. Secondly, though we utilized the standard FDA‐approved, clinically‐applied regimen of Physio‐Stim PEMF (maximum amplitude 1.19 mT, fundamental frequency 3.85 kHz, and duty cycle 25%) (PS, Orthofix, Inc), it is unclear if this is the optimal regimen to exert an effect on the IVD. Indeed, multiple studies^26^, ^27^, ^28^, ^29^, ^84^ have investigated the effect of varying PEMF signals (eg, waveform type, signal intensity, signal amplitude, pulse frequency, and treatment duration) on cell and tissue responses and some^26^, ^27^, ^28^, ^29^ have demonstrated differential effects based on regimen variation. Thirdly, unlike the clinical PEMF system—which is applied locally—we applied PEMF systematically to the rat. Therefore it is unclear the extent to which off‐target PEMF effects may contribute to the current findings. Lastly, this study is the first to investigate the effect of PEMF on IVD cells in their in vivo environment, including 3‐dimensional tissue structural elements and mechanical loading. However, we did not include a clinically relevant outcome measure (eg, differential changes in rat pain responses). To assess the possible therapeutic role of PEMF on disc degeneration—and the interaction between rat behavior and changes in inflammatory, anabolic, and catabolic factor expression—future studies should include a behavioral outcome, such as pain response.

## 9 CONCLUSION

We report the first in vivo demonstration that PEMF leads to the inhibition of acute inflammatory cytokines (IL‐6, IL‐β, and TNF‐α) 7 days after needle‐stab injury in the rat‐tail disc. However, a consistent effect of PEMF on acute changes in disc histology and anabolic and catabolic factor expression was not observed. The inhibitory effect of PEMF treatment on acute inflammatory cytokine expression in IVD cells may underlie its use as a potential therapy for disc degeneration‐associated inflammation and low back pain.

## AUTHOR CONTRIBUTION

All authors had substantial contributions to research design and/or the acquisition, analysis, or interpretation of data, were involved in drafting the manuscript and revising it critically, and read and approved the final submitted version of the manuscript.

## Supporting information


**FIGURE S1** Low, intermediate, and high histologic rating scale examples of rat caudal disc at day 4 and 7 after needle stab and PEMF treatment for needle stab (S) and needle stab + PEMF (SP) groups. Safranin O staining showing matrix production and nucleus pulposus structure; counterstained with Fast Green. A, Day 4 low needle stab (S). B, Day 4 intermediate needle stab (S). C, Day 4 high needle stab (S). D, Day 7 low needle stab (S). E, Day 7 intermediate needle stab (S). F, Day 7 high needle stab. G, Day 4 low needle stab +PEMF (SP). H, Day 4 intermediate needle stab +PEMF (SP). I, Day 4 high needle stab +PEMF (SP). J, Day 7 low needle stab +PEMF (SP). K, Day 7 intermediate needle stab +PEMF (SP). L, Day 7 high needle stab +PEMF (SP). AF, annulus fibrosus; NP, nucleus pulposus; CEP, cartilage endplate
