# Supplementary figures and images for: Pulsed electromagnetic fields reduce acute inflammation in the injured rat‐tail intervertebral disc
Source: JOR Spine. 2019 Dec 2;2(4):e1069. doi: 10.1002/jsp2.1069 (PMC6920683; doi:10.1002/jsp2.1069)

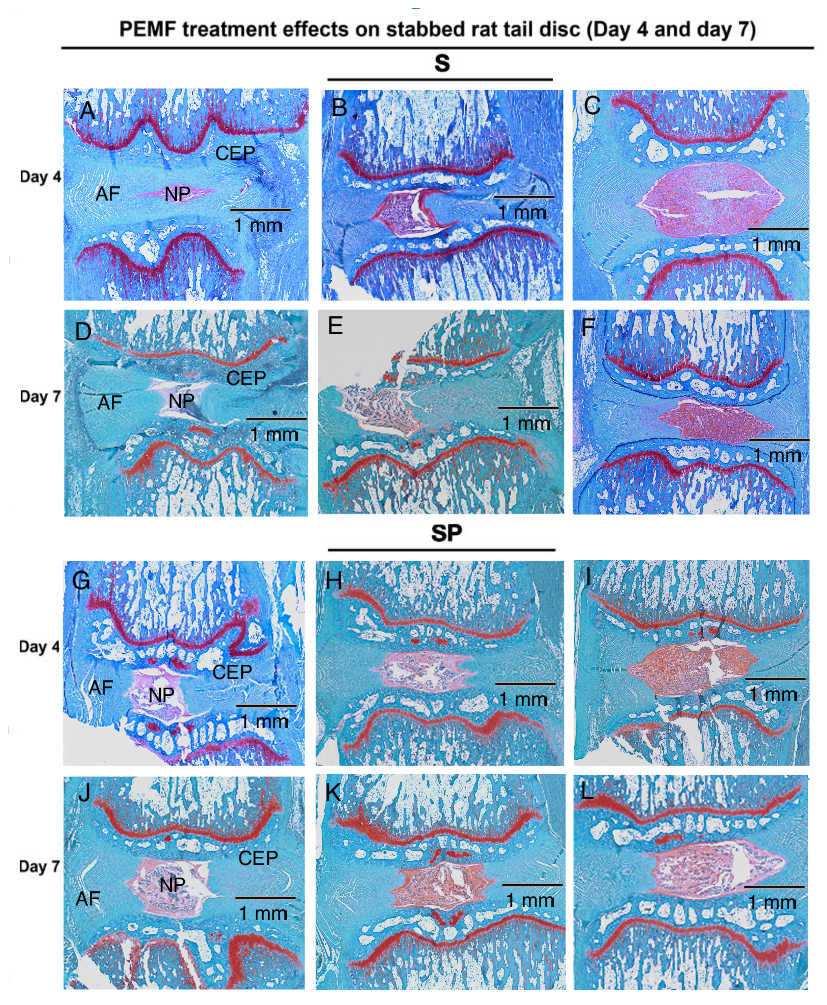

Supplement: Supplementary file 1 — FIGURE S1 Low, intermediate, and high histologic rating scale examples of rat caudal disc at day 4 and 7 after needle stab and PEMF treatment for needle stab (S) and needle stab + PEMF (SP) groups. Safranin O staining showing matrix production and nucleus pulposus structure; counterstained with Fast Green. A, Day 4 low needle stab (S). B, Day 4 intermediate needle stab (S). C, Day 4 high needle stab (S). D, Day 7 low needle stab (S). E, Day 7 intermediate needle stab (S). F, Day 7 high needle stab. G, Day 4 low needle stab +PEMF (SP). H, Day 4 intermediate needle stab +PEMF (SP). I, Day 4 high needle stab +PEMF (SP). J, Day 7 low needle stab +PEMF (SP). K, Day 7 intermediate needle stab +PEMF (SP). L, Day 7 high needle stab +PEMF (SP). AF, annulus fibrosus; NP, nucleus pulposus; CEP, cartilage endplate [file JSP2-2-e1069-s001.tif]
